# Supplementary figures and images for: Overexpression of Myo1e promotes albumin endocytosis by mouse glomerular podocytes mediated by Dynamin
Source: PeerJ. 2020 Mar 17;8:e8599. doi: 10.7717/peerj.8599 (PMC7083160; doi:10.7717/peerj.8599)

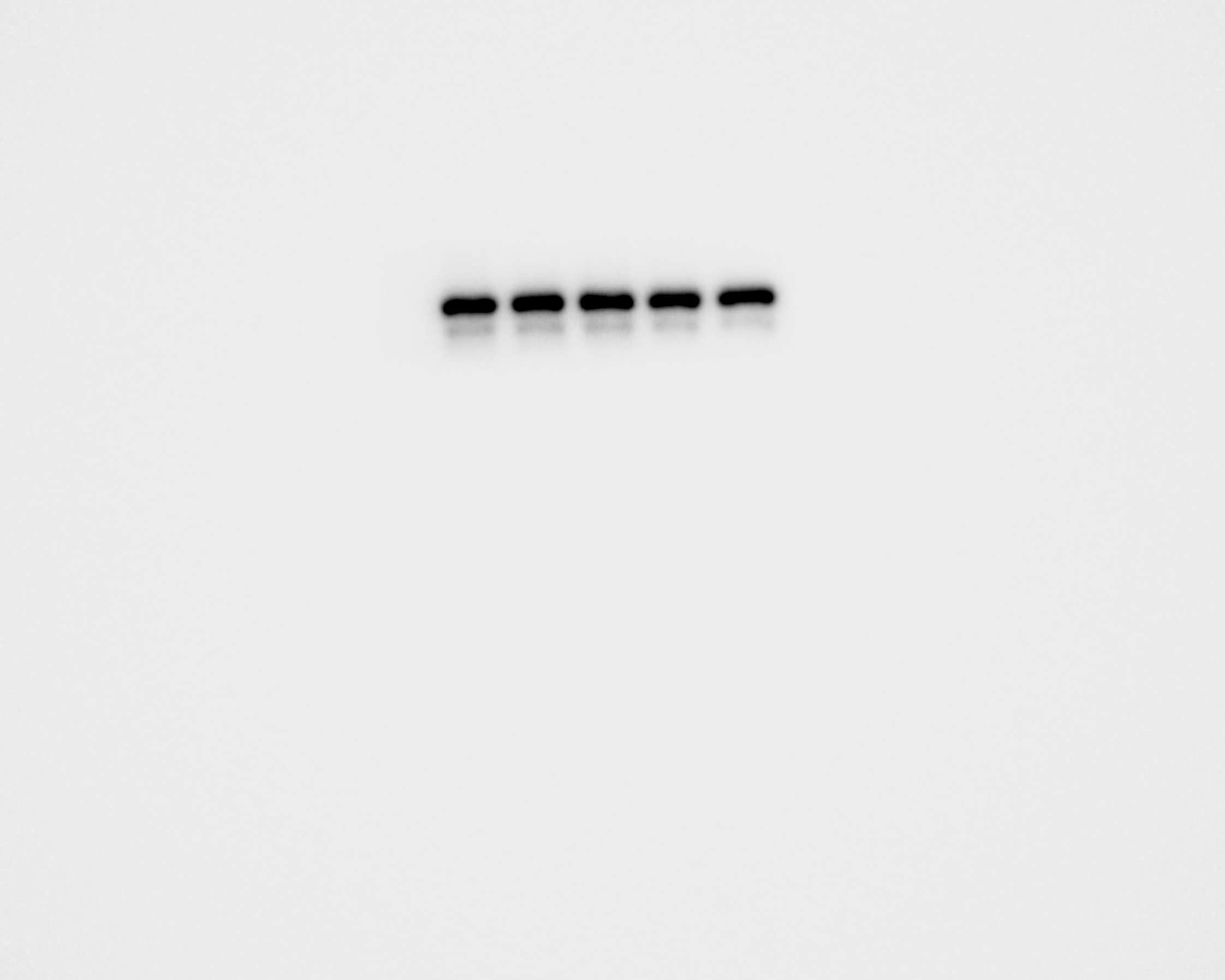

Supplement: Data S1 [file peerj-08-8599-s009.zip › Supplementary Data 4/GAPDH.tif]

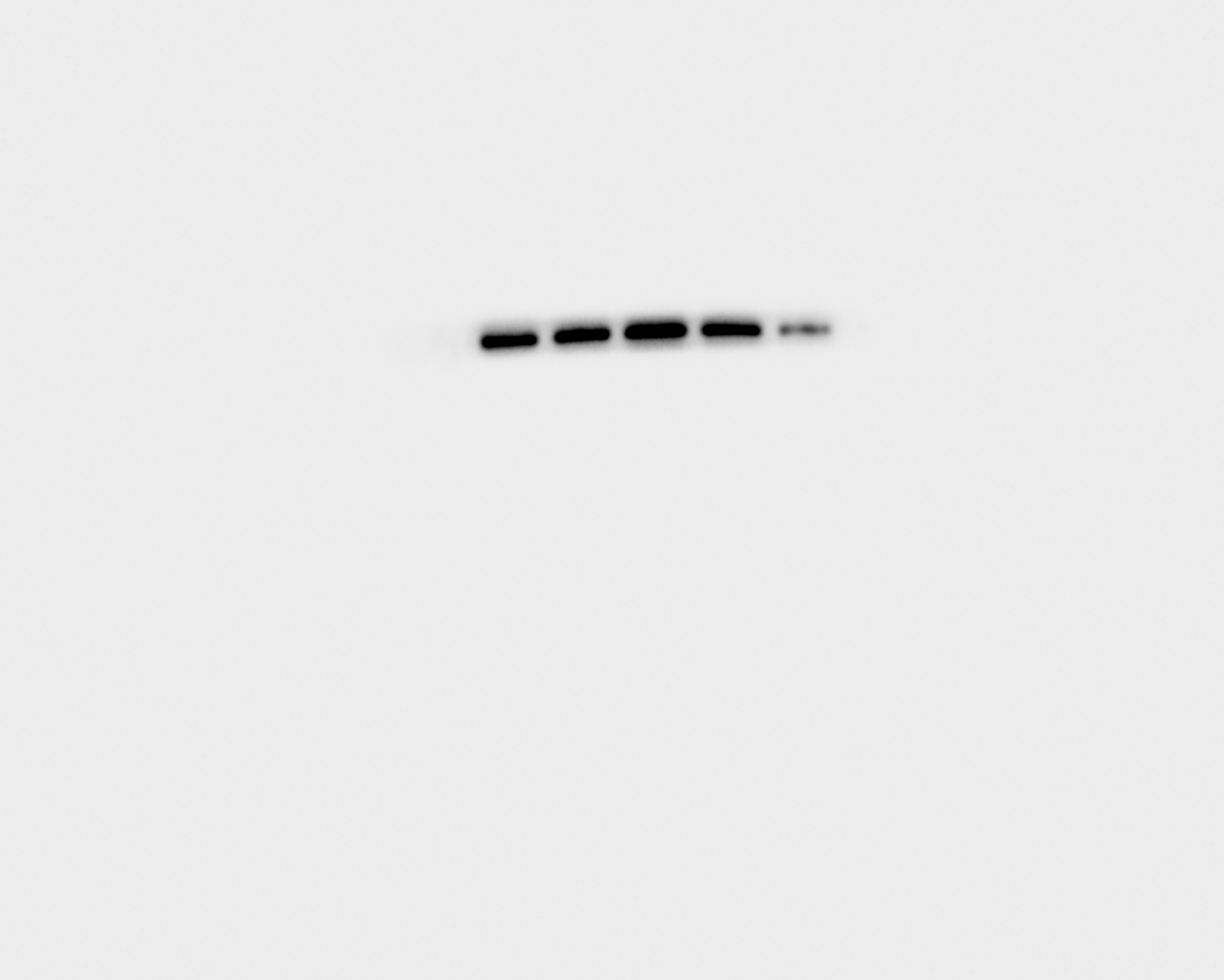

Supplement: Data S1 [file peerj-08-8599-s009.zip › Supplementary Data 4/Nephrin.tif]

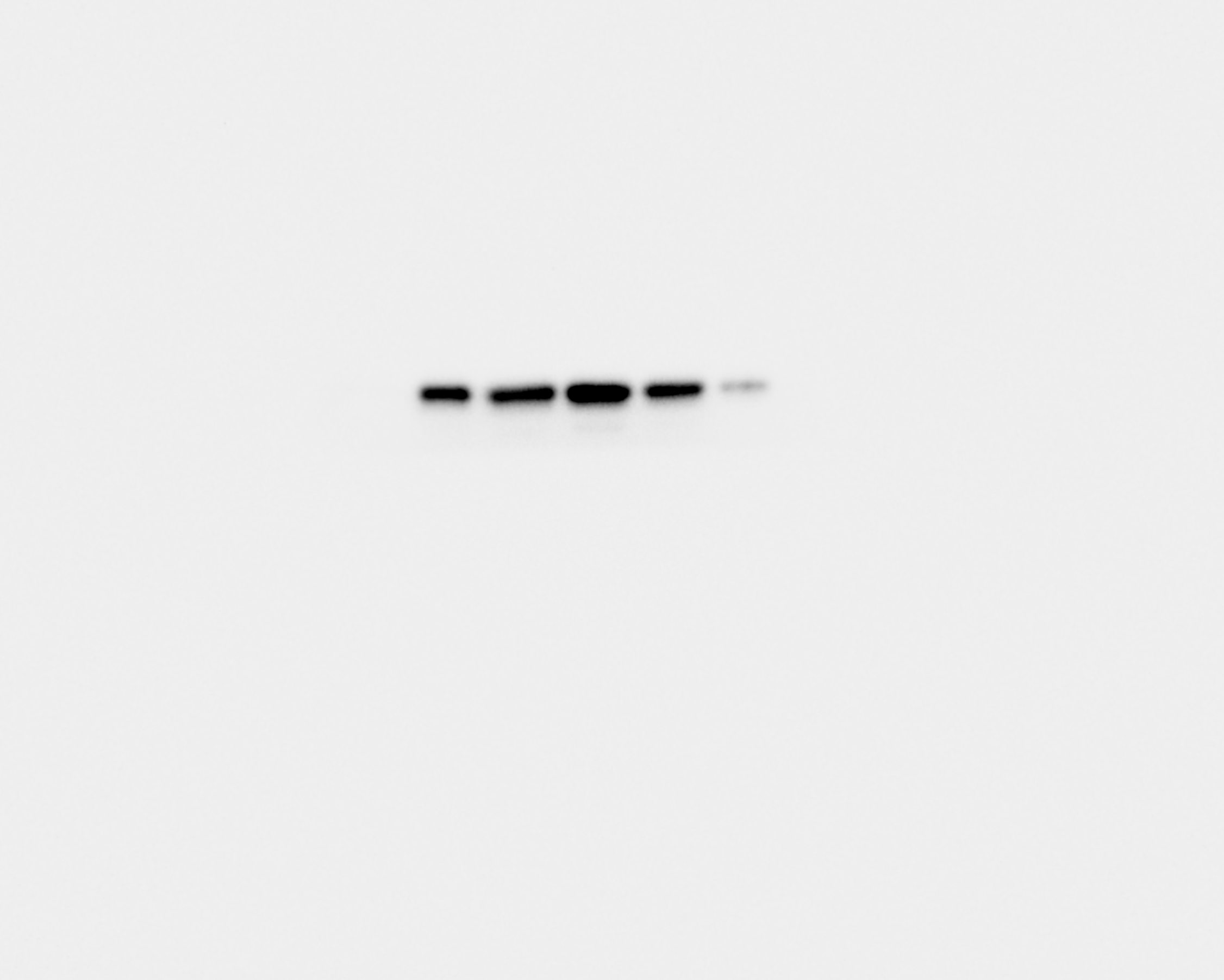

Supplement: Data S1 [file peerj-08-8599-s009.zip › Supplementary Data 4/Podocin.tif]
